# Unlocking the Nexus of Sirtuins: A Comprehensive Review of Their Role in Skeletal Muscle Metabolism, Development, and Disorders

**DOI:** 10.7150/ijbs.96885

**Published:** 2024-06-03

**Authors:** Bahareldin Ali Abdalla Gibril, Xinwei Xiong, Xuewen Chai, Qiao Xu, Jishang Gong, Jiguo Xu

**Affiliations:** Jiangxi Provincial Key Laboratory of Poultry Genetic Improvement, Institute of Biological Technology, Nanchang Normal University, Nanchang, 330032, China.

**Keywords:** sirtuins, skeletal muscle metabolism, development, disorders

## Abstract

The sirtuins constitute a group of histone deacetylases reliant on NAD^+^ for their activity that have gained recognition for their critical roles as regulators of numerous biological processes. These enzymes have various functions in skeletal muscle biology, including development, metabolism, and the body's response to disease. This comprehensive review seeks to clarify sirtuins' complex role in skeletal muscle metabolism, including glucose uptake, fatty acid oxidation, mitochondrial dynamics, autophagy regulation, and exercise adaptations. It also examines their critical roles in developing skeletal muscle, including myogenesis, the determination of muscle fiber type, regeneration, and hypertrophic responses. Moreover, it sheds light on the therapeutic potential of sirtuins by examining their impact on a range of skeletal muscle disorders. By integrating findings from various studies, this review outlines the context of sirtuin-mediated regulation in skeletal muscle, highlighting their importance and possible consequences for health and disease.

## 1. Introduction

Serving as the largest organ in mammals and accounting for 40-50% of adult-healthy human body mass, skeletal muscle is integral to whole-body metabolism, crucial for insulin-mediated glucose disposal and lipid catabolism, undergoing intricate molecular, cellular, and systemic regulation essential for movement, metabolism, and overall health 1,2. Sirtuins, a family of evolutionarily conserved deacetylases that rely on NAD^+^, have emerged as critical modulators in intercellular balance and stress response pathways. Their intricate involvement spans various cellular processes, including metabolism, stress resistance, genomic stability, DNA repair, aging, longevity, and disease 3-5. In the context of skeletal muscle, the roles of sirtuins have garnered considerable attention owing to their involvement in metabolic regulation, developmental processes, and their putative implications in numerous muscular disorders. Understanding the influence of sirtuins in skeletal muscle biology holds promise for deciphering fundamental physiological mechanisms and unveiling potential therapeutic avenues. This review aims to consolidate current knowledge regarding the impact of sirtuins on skeletal muscle metabolism, development, regeneration, and disorders, offering a comprehensive perspective on their roles and implications in the complex environment of skeletal muscle biology.

## 2. Sirtuin Family

### 2.1 Structure and classification

**Figure 1:**
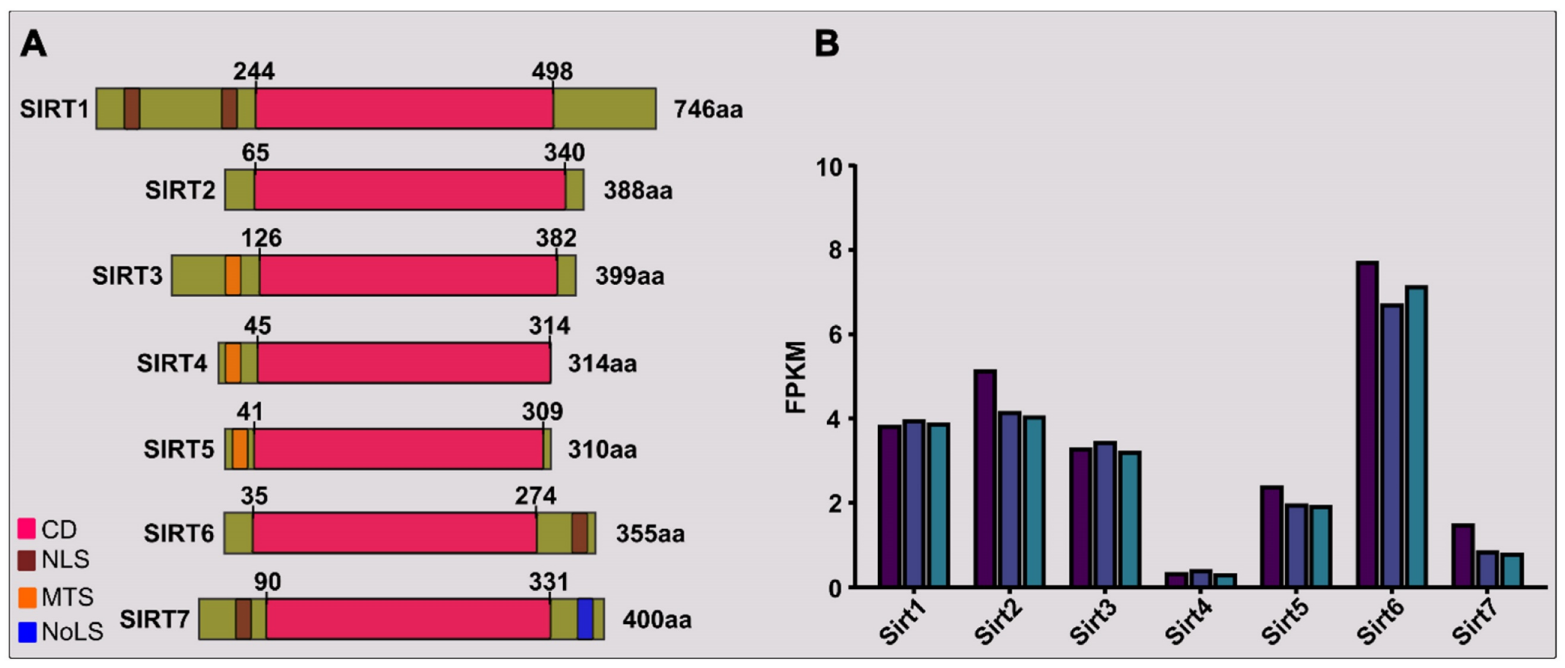
Structure and expression of sirtuins in primary skeletal myoblasts. (A) Seven members of mammalian (human) sirtuins are categorized with details such as catalytic domain (CD), nuclear localization sequence (NLS), mitochondria-targeting sequence (MTS), nucleolar localization sequence (NoLS), amino acid (aa) length, and positions. The illustration highlights the relative proportions of N-terminal and C-terminal extensions. (B) SIRT gene expression variations in primary chicken embryonic myoblasts are depicted through RNA-sequencing results (Bahareldin Ali Abdalla Gibril, unpublished). RNA samples, obtained under institutional guidance (approval no: SYXK(YUE)2014-0136), originated from primary myoblasts (3 samples) isolated from the leg muscle of 10-day-old chicken embryos. Each colored bar in the illustration corresponds to a distinct sample, with values indicating fragments per kilobase of transcripts per million (FPKM).

Class-III HDACs (histone deacetylases) include the sirtuin family, which is homologous to the yeast protein known as Sir2. This family encompasses a set of evolutionarily conserved proteins present in various organisms, from bacteria to humans. These proteins are characterized by their dependence on NAD^+^ for their enzymatic activities and play critical roles in regulating multiple cellular processes. In mammals, there exist seven sirtuin proteins named SIRT1 to SIRT7 (Figure 1A), each exhibiting unique subcellular localizations, functions, and enzymatic activities. The distribution of sirtuins across different cellular compartments is as follows: SIRT1 and SIRT6 are primarily in the nucleus, whereas SIRT2 mainly resides in the cytoplasm; however, SIRT1 and SIRT2 may be seen in the cytosol and nucleus, respectively. SIRT3/4/5 exists in mitochondria. SIRT7 is primarily located in the nucleolus, as shown in Table 1.

### 2.2 Enzymatic activities

Sirtuin enzymes utilize NAD^+^ as a cofactor to carry out enzymatic activities, such as deacetylating or deacylating various substrates (see Table 1). Research indicates that sirtuins exhibit diverse enzymatic activities, encompassing different protein modifications. For instance, SIRT4 functions primarily as an ADP-ribosyltransferase 6 but also possesses deacetylase 7, deacylase 8, and lipoamidase 9 capabilities. SIRT5, while lacking significant deacetylase activity, serves as a mitochondrial-lysine demalonylase, desuccinylase, and deglutarylase 10. Despite its comparatively low deacetylase activity, SIRT6 exhibits ADP-ribosyltransferase activity 11. Furthermore, SIRT6 efficiently eliminates myristoyl groups 12,13.

### 2.3 Expression and function in skeletal muscle

In a preliminary RNA-sequencing study of proliferating primary myoblasts from healthy 10-day-old chicken embryonic leg muscle, we observed significant variability in the expression of *Sirt* transcripts. *Sirt6* emerged as the most abundant among the seven family members, with *Sirt2*, *Sirt1*, and *Sirt3* showing relatively higher expression levels. Conversely, *Sirt4* exhibited the lowest abundance (Figure 1B). These findings offer insight for prioritizing studies on sirtuins in muscle developmental biology.

Sirtuins have attracted scientific attention for their crucial roles in metabolism, DNA repair, stress responses, and gene expression, impacting various cellular processes 4,5,14. Their relevance extends to skeletal muscle health, with SIRT1, SIRT2, SIRT3 and SIRT6 implicated in muscle development, metabolism, and the body's response to exercise-induced stress. Additionally, sirtuin activators have been identified to enhance skeletal muscle formation.

## 3. Sirtuins in Skeletal Muscle Metabolism

**Figure 2:**
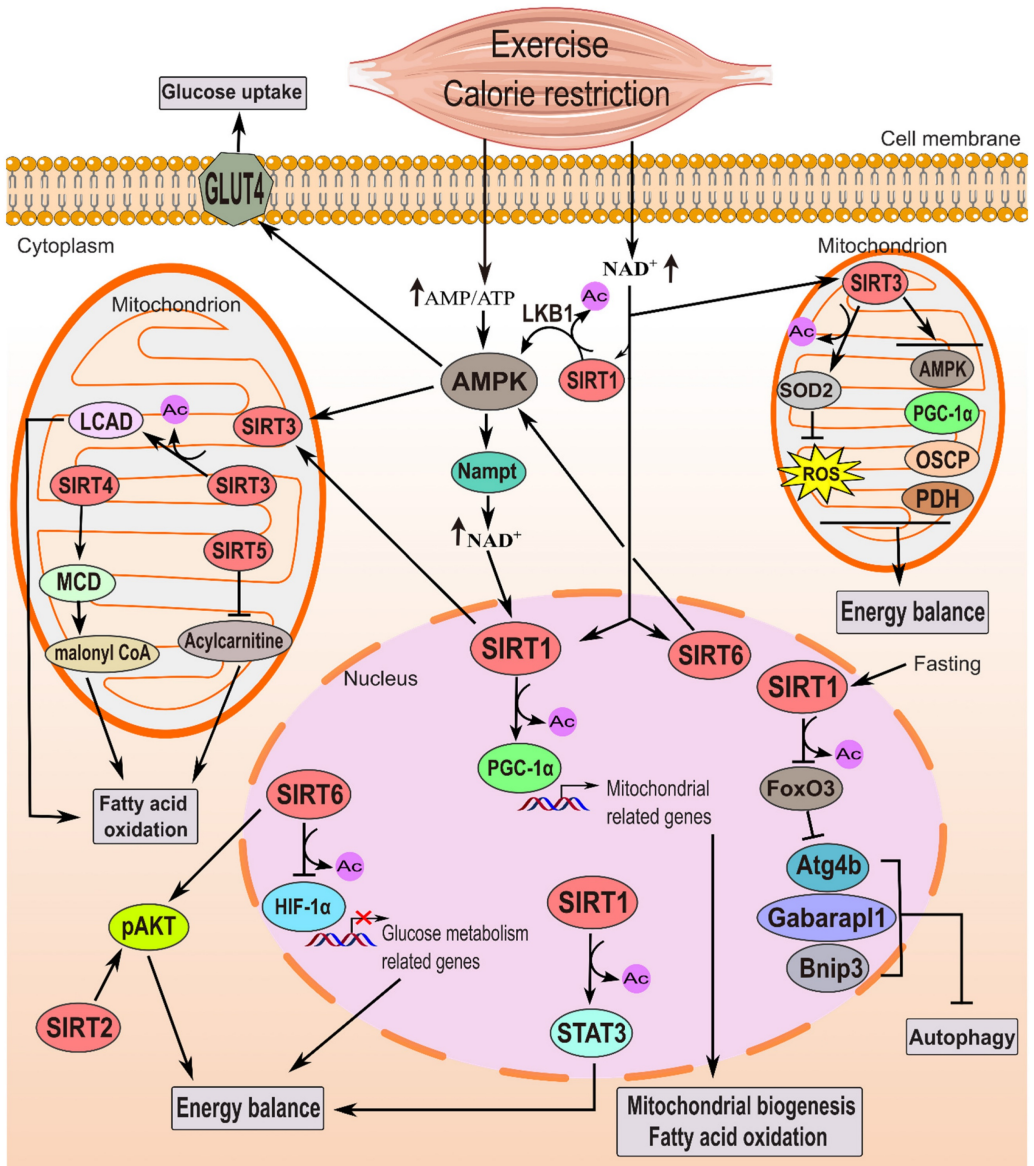
The function of sirtuins in governing skeletal muscle metabolism. Energy-deprived conditions, such as exercise and CR, elevate the AMP/ATP ratio, activating AMPK. This energy deficiency also boosts NAD^+^ levels, activating sirtuin's deacetylase function. Nampt is presumed to facilitate NAD^+^ production. AMPK enhances glucose uptake via GLUT4 activation and, in tandem with SIRT1, stimulates PGC-1α through deacetylation and phosphorylation, promoting FAO and mitochondrial biogenesis. There is cross talk as AMPK raises NAD^+^ levels, while SIRT1 activates AMPK via LKB1 deacetylation. SIRT3 and SIRT6 also activate AMPK. SIRT1 additionally influences energy homeostasis by activating Stat3 and inhibiting autophagy-related genes during fasting. FoxO3 deacetylation and inhibition by SIRT1 lead to a decrease in autophagy in muscles during 48 hours of fasting 61. In skeletal muscle, SIRT2 and SIRT6 improve insulin sensitivity, particularly under high-fat diets or insulin resistance, by enhancing p-AKT activation. SIRT6 also regulates energy balance by inhibiting HIF-1α. In mitochondria, SIRT3 enhances glucose metabolism, decreases ROS production via SOD2 stimulation, and promotes fatty acid oxidation by activating LCAD. SIRT4 activates MCD, while SIRT5 inhibits acylcarnitine, regulating fatty acid oxidation.

Skeletal muscle metabolism, crucial for energy balance, is dynamically regulated by sirtuins. These proteins play a key role in controlling skeletal muscle's metabolic processes, impacting mitochondria biogenesis, glucose uptake, fatty acid oxidation, autophagy, and exercise adaptations (Figure 2 and Table 2).

### 3.1 Glucose uptake regulation by sirtuins within skeletal muscle organ

Glucose uptake is crucial for energy metabolism in skeletal muscle, especially during increased energy demands like exercise or fasting. Sirtuins, notably SIRT1, SIRT2, SIRT3, and SIRT6, play significant roles in regulating this process. SIRT1, for instance, affects insulin-stimulated glucose transport in muscles, particularly under conditions of limited glucose availability 15. Caloric restriction (CR) enhances SIRT1 deacetylase activity and is associated with increased insulin-induced glucose absorption and phosphoinositide 3-kinase (PI3K) signaling in skeletal muscle 16. This effect is nullified in mice with impaired SIRT1 deacetylase activity specifically within their skeletal muscle tissues, suggesting its crucial role in enhanced insulin sensitivity during CR 16. SIRT1 also deacetylates and inactivates Stat3, impacting insulin signaling and PI3K activity in skeletal muscle 16. SIRT1 gain-of-function leads to increased basal phosphorylated AKT (p-Akt) levels, indicating potential improvements in insulin sensitivity 17.

Furthermore, studies show that SIRT1 mediates the impact of metabolic changes on regulating H4K16ac and myogenic differentiation-1 (MyoD) expression in muscle satellite cells 18. Endothelial SIRT1 deficiency enhances skeletal muscle insulin sensitivity through thymosin beta-4 (Tβ4)-mediated Akt signaling 19. AMPK, a key energy sensor, enhances glucose uptake and can be activated by various stimuli, including caloric restriction, fasting, and specific compounds like AICAR, metformin, and resveratrol 20-24. SIRT1 and AMPK exhibit a reciprocal interaction, where each can activate the other 25-27.

SIRT2 and SIRT3 also play crucial roles in skeletal muscle glucose metabolism. *Sirt2* knockout reduces insulin-induced glucose uptake and impairs insulin signaling, especially responding to a HFD 28. Conversely, SIRT3 deficiency results in impaired insulin signaling and elevated glucose levels, emphasizing its role in preserving insulin sensitivity 29. *Sirt3* full-body knockout mice exhibit impaired glucose oxidation in skeletal muscle due to decreased PDH activity. This impairment is characterized by an accumulation of lactate, pyruvate, and α-ketoglutarate metabolites, along with insulin's inability to suppress fatty acid oxidation. These metabolic changes are associated with hyperacetylation of the E1α subunit of PDH 30.

SIRT6 is essential for regulating glucose metabolism in skeletal muscle. SIRT6 negatively controls HIF-1α-dependent transcription, influencing glycolysis and glucose uptake even under normoxic conditions 31. Muscle-specific *Sirt6* knockout decreases insulin sensitivity and glucose homeostasis, emphasizing its regulatory role in skeletal muscle energy balance 32. *Sirt6* knockout mice exhibit fatal hypoglycemia due to increased glucose uptake, while SIRT6 overexpression enhances insulin sensitivity and glucose uptake 3,33. The apparent contradiction in glucose uptake effects observed with *Sirt6* knockout versus overexpression highlights the nuanced role of SIRT6 in glucose metabolism. The potential use of SIRT6 inhibitors for antidiabetic treatment 34 requires further investigation into the specific mechanisms involved and the potential therapeutic targets within glucose regulation pathways.

### 3.2 Influence of sirtuins on fatty acid oxidation within muscle cells

Skeletal muscle's fatty acid oxidation (FAO) is essential for lipid metabolism, particularly during heightened energy demands such as fasting or exercise. Fatty acid synthase facilitates cytoplasmic fatty acid synthesis. At the same time, FAO occurs in the mitochondrial matrix via β-oxidation, producing acetyl-CoA for ATP (adenosine triphosphate) synthesis in the tricarboxylic acid cycle (TCA). Sirtuins, notably SIRT1/3 and SIRT5, significantly influence FAO in muscle cells (Figure 2 and Table 2). SIRT1 interacts with transcription factors like PGC-1α to promote fatty acid breakdown and energy production by upregulating FAO enzyme genes 35. It's essential for initiating FAO in low-glucose environments by deacetylating and activating PGC-1α 21,35. Additionally, SIRT1 can activate AMPK 36, further stimulating FAO and inhibiting hepatic fatty acid production 37. SIRT3, on the other hand, maintains mitochondrial health by deacetylating and activating enzymes involved in FAO, promoting efficient FAO within mitochondria 38,39. Its deficiency leads to impaired FAO and energy production during fasting 40. SIRT4 negatively regulates fat oxidation, as observed *in vitro* and in *Sirt4* knockout mice, which exhibit increased FAO 7,41. However, SIRT3 and SIRT5 positively regulate FAO 39,42,43. SIRT5 ablation results in impaired β-oxidation, leading to the accumulation of medium- and long-chain acylcarnitines, crucial for transporting fatty acids into mitochondria 42,44. Overall, SIRT1, SIRT3, and SIRT5 coordinate multiple pathways to enhance FAO in muscle cells, crucial for cellular energy homeostasis during metabolic stress. More investigation is required to fully understand the broader impact of SIRTs on skeletal muscle metabolism, including their influence on glucose uptake and mitochondrial function.

### 3.3 Sirtuins' impact on skeletal muscle mitochondrial biogenesis and function

Mitochondria, known as the cell's "powerhouses," produce ATP, essential for cellular energy. Sirtuins, particularly SIRT1, SIRT3 and SIRT4, significantly influence mitochondrial biogenesis and function within skeletal muscle, which is vital for energy metabolism and cellular health. SIRT1 has a central role in regulating mitochondrial biogenesis, with its activity enhanced by increasing NAD^+^ levels, often augmented by CR 45. It interacts with and deacetylates PGC-1α, promoting mitochondrial biogenesis and function 35,46. FoxO1/3 are specific SIRT1 targets, suggesting SIRT1's role in mitochondrial biology regulation 47. Loss or reduction of SIRT1 activity leads to a decline in mitochondrially encoded oxidative phosphorylation (OXPHOS) genes and components, indicating mitochondrial dysfunction 48. Conversely, SIRT1 overexpression enhances mitochondrial function and biogenesis, particularly under HFD conditions, highlighting its regulatory role 17,49. SIRT1 activators also promote mitochondrial capacity, demonstrating their potential to enhance skeletal muscle function 50,51. SIRT3, a mitochondrial deacetylase, regulates mitochondrial activity by deacetylating specific proteins, including superoxide dismutase-2 (SOD2), mitigating oxidative damage and preserving mitochondrial integrity 52,53. *Sirt3* knockout leads to decreased mitochondrial markers and impaired AMPK and CREB phosphorylation, indicating mitochondrial dysfunction 54. SIRT3 also influences mitochondrial ATP levels through deacetylation of ATP synthase proteins (such as OSCP), which is crucial for energy homeostasis 55. SIRT4 has emerged as a crucial controller of mitochondrial phenotype and function in skeletal muscle cells. Through its influence on mitochondrial fusion proteins, including Mfn1 and OPA1, as well as metabolic pathways related to FAO, SIRT4 significantly contributes to the modulation of energy metabolism. Moreover, SIRT4 shapes mitochondrial morphology towards elongation, ultimately enhancing oxidative capacity within the cell 56. *Sirt4* knockout mice exhibiting impaired β-oxidation in the liver and skeletal muscles 42. *Sirt6* knockout in monkeys leads to immature mitochondria in fast-twitch muscle fibers 57. *Sirt7* knockout decreases protein levels of OXPHOS complexes in skeletal muscle, requiring additional research 58. In summary, sirtuins orchestrate a complex network regulating mitochondrial function in skeletal muscle, which is crucial for maintaining cellular energy balance and overall metabolic health. Further research is needed to elucidate their precise mechanisms and therapeutic potential in combating metabolic disorders.

### 3.4 Role of sirtuins in modulating autophagy and mitophagy in muscle cells

Autophagy, the cellular process responsible for breaking down and recycling damaged cellular components, is crucial for skeletal muscle health, development, and adaptation to exercise. While generally beneficial, dysregulation of autophagy can detrimentally impact muscle function. Sirtuins, notably SIRT1 and SIRT2, play significant roles in modulating autophagy. SIRT1 has diverse targets involved in autophagy regulation, including H4K16, autophagy-related (Atg) proteins, PGC-1α, and various transcription factors 16,59. Specifically, SIRT1 can deacetylate Atg proteins, including Atg7, Atg5, and Atg8/LC3, thereby promoting autophagy 60. SIRT1 acts as a negative regulator of autophagy by deacetylating H4K16, among others, while SIRT1 overexpression induces autophagy 60. During fasting, SIRT1 levels in mouse tibialis anterior (TA) muscles decrease significantly after two days, coinciding with the induction of autophagy genes such as Gabarapl1 (an Atg8 homologue), Atg4b and Bnip3. This decline in SIRT1 does not occur at earlier stages of fasting (12-24 hours) and is not observed in the liver or heart, suggesting muscle-specific regulation of SIRT1 in response to fasting. Upon 48 hours of fasting, electroporation of SIRT1 overexpression into the TA muscle of adult mice blocks the induction of autophagy genes Gabarapl1, Atg4b, and Bnip3 61 (Figure 2). This suggests that SIRT1 can inhibit autophagy, a process that, when overactivated, contributes to muscle wasting. Forkhead box O3 (FoxO3), a key regulator of autophagy in skeletal muscle, controls the transcription of autophagy-related genes such as LC3 and Bnip3 62. SIRT1 deacetylates the transcription factor FoxO3, responsible for the up-regulation of autophagy genes such as Gabarapl1, Atg4b, and Bnip3. This deacetylation leads to a reduction in FoxO3 activity, thereby inhibiting the expression of these autophagy genes 61. In endothelial cells, SIRT1 deficiency hampers autophagy, impacting insulin sensitivity 19. Treatment with resveratrol, a SIRT1 activator, restores autophagy-related gene expression and mitigates muscle injury 63,64. SIRT2 is pivotal in regulating skeletal muscle autophagy, particularly protecting against dexamethasone-induced muscle atrophy. It modulates autophagy-related gene expression, including LC3b and Beclin-1, thereby counteracting muscle loss triggered by excessive autophagy 65. SIRT2 deficiency disrupts the balance of acetylated FoxO1 interaction with Atg7, further affecting autophagy regulation. SIRT3 interacts with and deacetylates FoxO3 in heart cells, suggesting a potential role in regulating skeletal muscle autophagy 66. The influence of sirtuins on autophagy in muscle cells extends to the direct and indirect regulation of essential proteins and pathways like mTORC1 and AMPK, necessitating deeper exploration.

Age-related declines in mitochondrial function significantly impact skeletal muscle health. To maintain cellular health and performance, dysfunctional mitochondria are removed through mitophagy, a selective form of autophagy targeting damaged mitochondria for degradation 67-69. Many biomarkers of mitophagy are expressed in skeletal muscle, including PINK1, Parkin, BECN1 (Beclin-1), BNIP3, BNIP3L, FUNDC1, and mitofusin 2 (MFN2), reflecting the complex regulatory network governing mitochondrial quality control 69-73. Sirtuins such as SIRT1, SIRT2, SIRT3, and SIRT5 promote mitophagy in skeletal muscle, aiding in the maintenance of healthy mitochondria. They influence key regulators including PINK1, Parkin, BNIP3, Beclin-1, MFN2, and FoxOs 64,74-77. SIRT1 deacetylates MFN2, activating autophagy and mitophagy in liver cells 78. Resveratrol-induced mitophagy in C2C12 myoblast cells reduces damaged mitochondria and ROS levels, indicating therapeutic potential 64. Conversely, SIRT2-deficient mice exhibit defective mitophagy, with increased levels of PINK1, Parkin, P62 (also called sequestosome-1 or SQSTM1), and ubiquitin in brain tissues 74. Activation of SIRT3 is critical for inducing mitophagy under hypoxic stress, while inhibiting SIRT3 impairs mitochondrial quality control, reducing mitophagy-related proteins like PINK1 and Parkin in skeletal muscle 75. Activation of SIRT3 by AR-C17 (5-heptadecylresorcinol) improves mitochondrial function and mitophagy in obese skeletal muscle, increasing key autophagy and mitophagy proteins (Atg3/5 and Beclin-1, PINK1, and Parkin) in skeletal-muscle of HFD-fed mice and C2C12 cells through the SIRT3/FoxO3 pathway 76. In SIRT5-silenced C2C12 cells with the SIRT5 inhibitor MC3482, BNIP3 levels increased, indicating enhanced mitophagy commitment 77. SIRT5 inhibition also reduced MFN2 and OPA1 levels, favoring mitochondrial fission over fusion. Further exploration of sirtuin-mediated mechanisms of mitophagy could lead to novel therapeutic approaches for muscle-related disorders and age-related declines in skeletal muscle function.

### 3.5 Sirtuins' involvement in exercise-mediated metabolic adaptations

Exercise-induced metabolic adaptations in skeletal muscle are pivotal for optimizing energy utilization, maintaining metabolic health, and enhancing overall physical performance. Regular physical activity triggers beneficial changes, including upregulating NAD^+^-dependent sirtuins, specifically SIRT1, SIRT3, and SIRT6. Exhaustive exercise notably increases NAD^+^ levels in mouse tibialis anterior muscle within three hours post-exercise 79. Acute exercise swiftly elevates SIRT1 levels in both rodents and humans, peaking within two hours 80. Long-term treadmill training enhances SIRT1 and PGC-1 levels, bolstering antioxidant defenses in rat muscles 81. However, chronic voluntary running fails to alter muscle SIRT1 expression, suggesting intensity and time dependency 82.

Muscle-specific *Sirt1* knockout mice display reduced OXPHOS and diminished endurance in long-distance running, while SIRT1 overexpression in older mice enhances fatigue resistance 83,84. SIRT1 is crucial for maintaining exercise endurance and muscle strength, as evident from reduced treadmill running distance and muscle function in muscle-specific SIRT1-KO mice 85,86. SIRT1 activators like SRT1720 and resveratrol enhance mice's aerobic capacity and endurance performance by promoting oxidative muscle fibers and genes related to FAO 22,46. Fatty acids are essential in inducing muscle protein adaptations because they bind to peroxisome-proliferator-activated receptors 87. In rats, acute endurance exercise quickly (two hours) increased SIRT1 protein levels in the soleus muscle. Following a 14-days of endurance exercise, both PGC-1α and SIRT1 proteins rose along with key metabolic components 80. In human skeletal muscle, training using high-intensity intervals for six weeks boosted mitochondrial enzyme activities (28-36%) and PGC-1α protein levels by 16%, while also increasing SIRT1 activity 88. The absence of SIRT1 deacetylase activity within skeletal muscle remained unimpaired mitochondrial biogenesis in response to voluntary wheel running and acute exercise 89. This challenges the notion that SIRT1 serves as a primary controller of skeletal muscle's exercise-induced mitochondrial biogenesis. SIRT1's impact on energy metabolism during exercise involves modulating glucose uptake, mitochondrial biogenesis, and FAO 90. In response to exercise training, SIRT3 expression increases selectively, particularly in muscles undergoing training, such as the triceps 54. Exercise-induced mitochondrial biogenesis is supported by increased citrate synthase activity 54. SIRT3 levels are reduced in immobilized hindlimb muscles but significantly higher in master athletes, indicating a potential role in maintaining mitochondrial health crucial for efficient ATP production during exercise 52. SIRT6 expression is elevated in chronically exercised humans, positively influencing exercise performance and mitochondrial-oxidative-capacity 91. *Sirt6*-KO mice show weakened exercise performance and lowered energy expenditure, while SIRT6 activation increases exercise endurance, mimicking the effects of exercise 91.

### 3.6 Oxidative stress and inflammation

Oxidative stress and inflammation are contributing factors to various muscle disorders, which are often associated with or implicated in the aging process. SIRT1 and SIRT3 act as crucial regulators, mitigating oxidative damage and inflammation.

*Antioxidant defense by sirtuins:* Sirtuins, particularly SIRT1/3, orchestrate antioxidant defense mechanisms in skeletal muscle. A potent SIRT1 activator, SRT2104 enhances mitochondrial function and counters oxidative stress via reducing protein carbonylation and 4-HNE levels and increasing SOD2 92, while SIRT3 reduces ROS levels in mitochondria 29,53,93, which are crucial for maintaining muscle health. Studies in *Sirt1* and *Sirt3* knockout mice underscore their significance in mitigating oxidative stress 29,53,94. SIRT2 may reduce oxidative stress via the Nrf2/FoxO3 pathway in skeletal muscle 95, which requires future study.

*Role of sirtuins in inflammation:* Sirtuins play a pivotal anti-inflammatory role in skeletal muscle. They suppress transcription factors that promote inflammation, such as HIF-1α, STAT3, and NF-κB 31,96, attenuating inflammatory cytokine expression. Sirt1-activating compounds demonstrate promising anti-inflammatory effects in muscle tissues 97.

*Interaction with signaling pathways:* Sirtuins interact with various signaling pathways to regulate inflammation. For instance, SIRT1's interaction with AMPK highlights its role in mitigating inflammation during muscle regeneration 48,98, emphasizing the multifaceted functions of sirtuins in muscle health. Future studies should focus on understanding their deeper mechanisms which provides avenues for therapeutic interventions targeting muscle disorders associated with aging and inflammation.

## 4. Sirtuins in Skeletal Muscle: Exploring their Role in Development and Regeneration

### 4.1 Contribution of sirtuins to myogenesis

Myoblast proliferation and differentiation, collectively known as myogenesis, are essential for skeletal muscle growth, development, and maintenance. During embryonic development, myoblast proliferation contributes to initial muscle tissue formation, while in adulthood, it supports muscle fiber growth in response to exercise or injury 99. Myoblasts differentiate into mature muscle cells by activating myogenic regulatory factors such as Myf5, MyoD, myogenin, Myf6, and paired box proteins PAX3 and PAX7, which drive muscle cell maturation through downstream gene expression.

Sirtuins, particularly SIRT1 and SIRT2, influence myoblast proliferation. *In vitro* studies using mouse C2C12 muscle cells show that SIRT1 and SIRT2 promote myoblast proliferation 100-103. However, treatment with SIRT1 activators like SRT2104 or resveratrol inhibits proliferation, while SIRT1 inhibition enhances proliferation, indicating a SIRT1-dependent mechanism 92,102. Conflicting data may arise from variations in experimental conditions, concentrations, or methodologies, necessitating further research for clarification. SIRT1 enhances muscle cell proliferation by regulating cell cycle regulators, such as inhibiting p21WAF/CIP1 or increasing p27Kip1 expression, and activating the Wnt signaling pathway 100,101. Similarly, SIRT2 achieves this effect by activating the ERK1/2 pathway 103. Treatment of primary myoblasts with the SIRT2 inhibitor AGK2 increased Myf5 expression 104. Additionally, the lncRNA Sirt-1AS, derived from the antisense strand of the SIRT1 locus, promotes muscle cell proliferation by inducing cyclin expression 105.

SIRT1 and SIRT3 also influence myoblast differentiation, with SIRT1 showing decreased expression during C2C12 differentiation. SIRT1 overexpression negatively affects myogenesis by downregulating myogenin, MEF2C, myosin heavy chain, and other differentiation-related genes 106,107. Muscle-specific Sirt1 knockout mice exhibit altered gene expression related to skeletal muscle development, notably upregulating the embryonic myosin-heavy-chain isoform Myh3 18. Myf5 expression increases early in differentiation, and MEF2 overexpression enhances MyoD expression, promoting differentiation 108. SIRT1 deacetylates MEF2 during muscle differentiation and interacts with PCAF/GCN5, deacetylating PCAF and MyoD 15,109. Interaction with the PGC-1 promoter is enhanced by MyoD 110. SIRT1 inhibitors such as nicotinamide (NAM), sirtinol, and M-15 activate muscle-specific enhancers 106. SIRT3 expression increases significantly during early differentiation, and its depletion inhibits myoblast differentiation, leading to decreased myogenin and MyoD expression and a lack of polynucleated myotubes 111.

NAD^+^ availability modulates SIRT1's deacetylase activity, with a reduction in the ratio of NAD^+^/NADH during muscle cell differentiation potentially affecting SIRT1 activity 106. SIRT1 mediates the impact of glucose restriction on skeletal muscle differentiation, as inhibition by NAM or sirtinol reverses differentiation impairment under glucose restriction 15,112. Myoblasts from mice with mutated SIRT1 genes show impaired differentiation under low glucose conditions, highlighting the necessity of SIRT1 for proper differentiation during glucose restriction 15. Glucose restriction triggers AMPK activation, leading to Nampt transcription, increasing intracellular NAD^+^ levels, and reducing NAM levels, subsequently activating SIRT1 and inhibiting skeletal muscle differentiation.

The role of SIRT1 in myogenesis seems contradictory based on different conditions (normal vs. glucose restriction), highlighting the complexity of cellular responses to metabolic stress and the nuanced regulation of gene expression and differentiation pathways by SIRT1. The involvement of AMPK and other metabolic signaling pathways likely modulates SIRT1 activity and its impact on myogenesis depending on the cellular environment. Further studies are needed to fully elucidate these intricate interactions and their implications for skeletal muscle biology under varying metabolic states.

### 4.2 Sirtuin regulation of determining muscle fiber type

**Figure 3:**
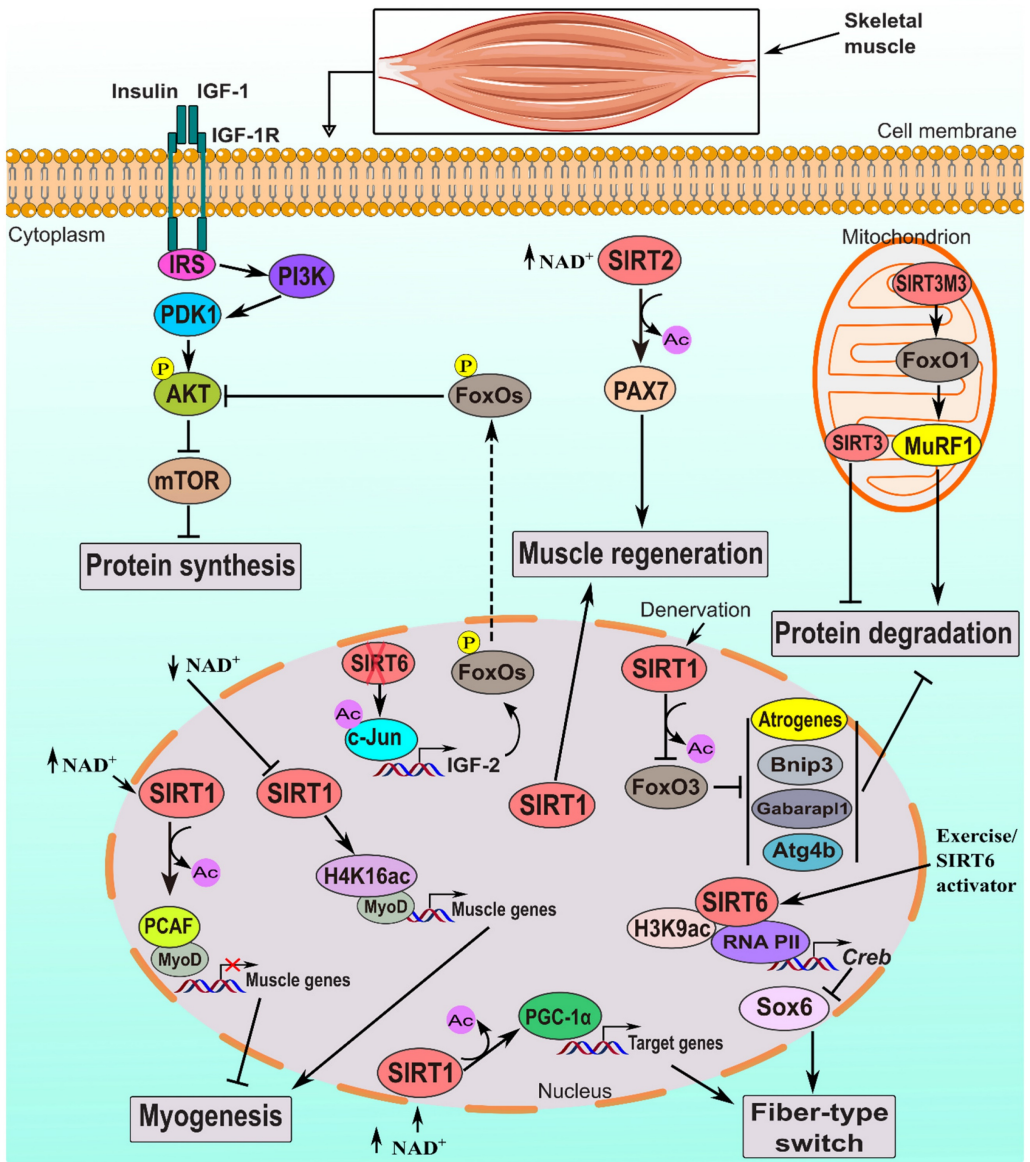
The multifaceted role of sirtuin in regulating skeletal muscle myogenesis, fiber-type switching, regeneration, and atrophy. Activated SIRT1 inhibits myogenesis by deacetylating MyoD and PCAF. Decreased NAD^+^ levels block SIRT1 activity, leading to global H4K16ac accumulation, which increases muscle gene expression by activating the MyoD promoter. SIRT1 induces fiber-type switching by deacetylating and activating PGC-1α. It also prevents protein degradation and atrophy (during conditions like denervation and fasting) by deacetylating and inactivating FoxO3, which suppresses atrogenes (Atrogin-1 and MuRF1) and autophagy genes (Gabarapl1, Atg4b and Bnip3) 61. Lack of SIRT1 impairs muscle regeneration. SIRT2 enhances muscle regeneration by deacetylating and activating PAX7. SIRT3 isoforms have contrasting effects on muscle atrophy: SIRT3M3 promotes atrophy by activating FoxO1, while SIRT3 isoform-1 inhibits atrophy, though the mechanisms are not fully understood. Activating SIRT6 influences Sox6 expression, regulating muscle fiber composition by promoting slow-twitch oxidative muscle fibers through downregulation of Sox6. Lack of SIRT6 hyperactivates IGF/PI3K/AKT signaling, leading to FoxOs transcriptional repression and decreased muscle atrophy. X in red: deletion.

Different types of fibers make up skeletal muscle, such as Type-I fibers that have an oxidative metabolism and a slow twitch, and Type-II fibers that have a glycolytic metabolism and a quick twitch. Each type has distinct contractile and metabolic properties. Sirtuins, particularly SIRT1, SIRT3, and SIRT6, play crucial roles in determining muscle fiber type (Figure 3 and Table 3). Slow-twitch muscles like the soleus exhibit higher expression levels of SIRT1, which preserves oxidative muscle fibers. SIRT1 regulates PGC-1α, contributing to fiber-type switching. Knockout of SIRT1 in mice leads to reduced oxidative phosphorylation and a shift toward glycolytic fibers 83,85,86. Overexpression of SIRT1 promotes slow-twitch fiber development and mitigates dystrophic characteristics, potentially through elevated PGC-1α levels 17,83. SIRT1 activators like resveratrol induce slow muscle fiber expression via the SIRT1-PGC-1α axis 113,114. SIRT3M3 is a shorter isoform of the mouse SIRT3 protein found in the mitochondria, nucleus, and cytosol. Despite being shorter, SIRT3M3 exhibits similar mitochondrial deacetylase activity compared to the full-length SIRT3 115. SIRT3 expression correlates with oxidative muscle types 54, and SIRT3M3 overexpression induces a transition to oxidative fibers, increases energy expenditure, and reduces muscle mass 107. Conversely, SIRT3 knockout accelerates lean mass loss and shifts muscle fibers toward fast-twitch types 116. SIRT6 regulates muscle fiber composition by promoting slow-twitch oxidative types through the downregulation of Sox6, a repressor of slow fiber genes 57,91. This regulatory role extends to enhancing mitochondrial oxidative capacity and exercise performance in mice 91.

### 4.3 Regulation of muscle regeneration by sirtuins

Skeletal muscle can regenerate and repair following injuries, a process vital for restoring muscle function and preventing long-term impairment. Central to this mechanism are satellite cells (SCs), a type of myogenic or muscle stem cell, which activate and differentiate, ultimately forming new muscle fibers 99,108. Critical regulators of muscle regeneration include SIRT1 and SIRT2 (Figure 3). SIRT1 significantly influences SC function, enhancing muscle regeneration by controlling proliferation and differentiation after injury or damage. Notably, the NAD^+^-SIRT1 axis regulates satellite cell activation, shifting metabolism from FAO to glycolysis. Reduced SIRT1 activity during this transition leads to increased acetylation of H4K16, affecting gene expression 18,117. Studies in Duchenne muscular dystrophy mouse models indicate that calorie restriction increases regenerative capacity in injured muscles and enhances satellite cell transplantation efficiency, likely through upregulating SIRT1 expression 118. Conversely, satellite cells lacking SIRT1 exhibit premature differentiation and impaired muscle regeneration in mice 18. Interventions elevating NAD^+^ levels, such as CR or nicotinamide riboside administration, enhance satellite cell functions, aiding muscle regeneration 37,118. Similarly, SIRT1 activation with resveratrol enhances satellite cells and muscle regeneration post-injury 119,120. Conversely, inhibiting SIRT1 impacts membrane resealing in myoblast cells, potentially affecting muscle regeneration mechanisms 85. Recent studies have implicated SIRT2 in muscle regeneration. SIRT2 interacts with Pax7, regulating its acetylation status and impacting the expression of PAX7 target genes such as Myf5. Additionally, SIRT2 modulates satellite cell asymmetric stem cell division, influencing muscle regeneration 104,121. These results imply that SIRT1/2 may be useful as a therapeutic target for disorders involving atrophy and reduced muscle regeneration. Further investigation into the interactions of SIRTs with AMPK and the roles SIRT3/6 in muscle regeneration is warranted 98,99.

### 4.4 Influence of sirtuins on skeletal muscle hypertrophy

Skeletal muscle hypertrophy, the enlargement of muscle fibers typically induced by resistance exercise or muscle overload, is crucial for lean muscle growth, muscle performance, and overall muscle health. Sirtuins, particularly SIRT1 and SIRT6, play significant roles in this process (Table 3). Research indicates that overload-induced hypertrophy in skeletal muscles of rats is linked to increased expression of SIRT1 but decreased SIRT3 level, which is implicated in mitochondrial degradation 122,123. SIRT1 also regulates myonuclear domain size in skeletal muscle fibers, where overexpression decreases domain size while inactivation increases it, with no significant impact on muscle force 124. Furthermore, SIRT proteins influence protein acetylation levels, which are closely linked with hypertrophy. SIRT1 overexpression leads to rapid fiber hypertrophy by activating FoxO1/3, inhibiting atrogens, and deacetylating FoxOs involved in muscle protein degradation. It also activates PGC-1α, inhibiting muscle wasting via suppressing the activity of NF-κB and FoxO3 61,125,126. Conversely, muscle-specific *Sirt1*-KO mice display impaired muscle development 18. Similarly, *Sirt6* knockout mice demonstrate decreased muscle mass and fiber thickness, with potential implications on muscle biology that deserve additional research. Notably, administration of nucleoside reverse-transcriptase inhibitors has shown a potential to ameliorate the effects of SIRT6 deficiency on muscle-related parameters 127.

## 5. Sirtuins in Skeletal Muscle Disorders

Sirtuins, especially SIRT1/6, play critical roles in regulating skeletal muscle disorders by modulating pathways related to protein degradation, muscle wasting, mitochondria, and inflammation (Table 4).

### 5.1 Muscle atrophy

Muscle atrophy, the muscle strength and mass loss, is influenced by various factors 2,128. Sirtuins, especially SIRT1, SIRT2, SIRT3, and SIRT6, play crucial roles in regulating muscle atrophy by modulating pathways related to protein degradation and muscle wasting. Studies show that SIRT1 deficiency exacerbates muscle atrophy, while its overexpression inhibits muscle wasting by suppressing critical atrogenes and reducing overall proteolysis 61,92.

Atrogenes, such as Atrogin-1 (MAFbx) and MuRF1, regulate muscle protein breakdown and are often upregulated in conditions causing muscle loss, like disuse, aging, or certain diseases. Resveratrol mitigates muscle aging and atrophy 129. SIRT1 also controls muscle cachexia associated with chronic diseases like cancer 130. Cachexia, often seen in cancer patients, is marked by significant weight loss, muscle wasting, anorexia, and weakness, involving increased muscle protein breakdown.

SIRT2 and SIRT3 play distinct roles in muscle atrophy. Inhibiting SIRT2 reduces muscle mass through autophagy-related protein regulation 65, while SIRT3 deficiency accelerates atrophy by affecting protein acetylation and mitochondrial function 116. SIRT3 reduces protein degradation in the AR100Q mouse model, used to study neurodegenerative diseases like SBMA or Kennedy's disease, where mutations in the androgen receptor (AR) gene cause toxic effects on skeletal muscle, leading to muscle weakness and neurodegeneration. SIRT3 achieves this by reducing protein acetylation in metabolic pathways such as the TCA cycle and fatty acid beta-oxidation 131. Conversely, SIRT3M3 enhances muscle protein degradation by upregulating FoxO1 expression and increasing MuRF1 expression 107. SIRT6 regulates muscle atrophy by modulating IGF/PI3K/AKT signaling and influencing the expression of atrogenes 132. It also plays a role in cancer-associated cachexia, preserving muscle mass and function while suppressing tumor growth 133. Understanding sirtuin function in skeletal muscle offers potential avenues for preventing and treating muscle-related illnesses.

### 5.2 Muscular dystrophy

Genetic diseases known as muscular dystrophies are characterized by increasing muscle weakening and degeneration, primarily due to gene mutations essential for muscle cell structure and function. Sirtuins, particularly SIRT1 and SIRT6, promise to mitigate muscular dystrophy (MD) effects by improving muscle function and reducing degeneration in preclinical models. In Duchenne MD (DMD), mutations in the DMD gene lead to dystrophin protein absence, crucial for muscle integrity. In a study using the mdx mouse model of DMD, previously injured tibialis anterior muscles from calorie-restricted mice exhibited enhanced regenerative capacity compared to controls, attributed to elevated NAD^+^ levels and SIRT1 upregulation 118. Skeletal muscle-specific *Sirt1*-KO mice demonstrate a somewhat dystrophic phenotype, whereas mdx mice with SIRT1 overexpression have improved pathology and enhanced exercise capacity 83,85. Resveratrol, also enhances characteristics of muscular dystrophy in mdx mice 63,64. In myofibers, SIRT6 deacetylates H3K56ac, which inhibits the production of utrophin. In the mdx mouse, utrophin decreases disease by stabilizing the sarcolemma when dystrophin is not present. SIRT6 inactivation in mdx mice reduces myofiber disruption, improving muscle function 134. These findings suggest targeting SIRT1 and SIRT6 as potential therapeutic avenues for DMD, opening new paths for effective treatments.

### 5.3 Metabolic disorders affecting muscle (insulin resistance and diabetes)

Metabolic disorders disrupt muscle function by impacting metabolism. Sirtuins, including SIRT1, SIRT2, SIRT3, and SIRT6, play crucial roles in regulating cellular metabolism, and their dysregulation contributes to insulin resistance and diabetes, notably affecting skeletal muscle. Insulin resistance is correlated with a decrease in SIRT1 levels in muscle tissue in type 2 diabetes 135-137. Insulin resistance correlates with diminished mitochondrial presence in skeletal muscle 138. SIRT1 and resveratrol enhance insulin sensitivity by suppressing the expression of PTP1B, a crucial enzyme involved in insulin signaling and glucose metabolism regulation. This suppression prevents tyrosine dephosphorylation on the insulin receptor and substrate 136. In C2C12 cells, SIRT1 overexpression improves insulin sensitivity under high glucose conditions 49, while *in vivo*, insulin resistance caused on by a high-fat diet is reduced by modest SIRT1 overexpression, which improves mitochondrial performance and lowers oxidative stress 137. Electroacupuncture treatment in mice improves insulin sensitivity by upregulating SIRT1 expression in skeletal muscle 139. Conversely, SIRT2 deletion induces muscle insulin resistance, while inhibiting SIRT2 improves insulin sensitivity in insulin-resistant muscle cells 28,140. SIRT3 expression reduction contributes to insulin resistance, and its overexpression enhances glucose uptake and reduces ROS production in insulin-resistant muscle cells 29,141,142. Enhanced SIRT6 levels defend against diet-induced type 2 diabetes by improving insulin sensitivity in muscle and liver, while inhibiting SIRT6 improves glucose tolerance 33,34. These findings suggest that targeting sirtuins could offer therapeutic strategies for managing insulin resistance and diabetes. Further research is needed to explore optimal methods for modulating SIRT6 in type 2 diabetes.

## 6. Conclusion and Future Perspectives

In conclusion, skeletal muscle undergoes significant metabolic adaptations in response to exercise, with sirtuins playing pivotal roles in orchestrating these biological events. SIRT1, SIRT2, SIRT3, and SIRT6 are key regulators of mitochondrial biogenesis, fatty acid oxidation, and antioxidant defense mechanisms, crucial for optimizing energy utilization and enhancing physical performance. Additionally, sirtuins play critical roles in skeletal muscle development, including myogenesis, determination of muscle fiber type, regeneration, and hypertrophic responses. Moreover, the therapeutic potential of sirtuins in addressing various skeletal muscle disorders is underscored by their impact on muscle health and function. To leverage the full-potential of sirtuins for therapeutic interventions, there is a compelling need to develop and rigorously test small molecule SIRT activators or inhibitors tailored for specific muscle disorders. Such compounds could modulate sirtuin activity to promote mitochondrial health, improve energy metabolism, and mitigate muscle dysfunction associated with aging and disease. Furthermore, NAD^+^ boosting therapies, which enhance cellular NAD^+^ levels and thereby activate sirtuins, hold promise for combating age-related declines in muscle function. By augmenting NAD^+^ availability, these therapies could enhance sirtuin-mediated metabolic adaptations and promote muscle health during aging and in muscle disorders. Future investigations should focus on elucidating the intricate mechanisms underlying sirtuin-mediated metabolic adaptations induced by exercise, and explore innovative therapeutic strategies to enhance the benefits of exercise on muscle health. Research efforts should also prioritize addressing age-related declines in muscle function through targeted interventions harnessing the regulatory potential of sirtuins. In summary, leveraging the regulatory capabilities of sirtuins through the development of specific modulators and NAD^+^ boosting therapies represents a promising avenue for advancing therapeutic approaches to enhance mitochondrial function, optimize energy metabolism, and mitigate age-related muscle loss, ultimately contributing to overall health and well-being.

## Figures and Tables

**Table 1:** Mammalian sirtuins: Key biological attributes in skeletal muscle function.

SIRTs	Primary localization	Activity	Targets	Biological functions	Tested activators	Tested inhibitors
SIRT1	Nucleus	Deacetylase	PCAF/MyoD, MEF2	Myogenesis	SRT2104/SRT1720/Resveratrol/NMN/Myricanol	EX527/NAM/Sirtinol/M-15/6-chloro-2,3,4,9-tetra-hydro-1-*H*-carbazole-1-carboxamide
			PGC-1α, Stat3	Energy homeostasis		
			H4K16ac	Chromatin modification		
			p53	Cellular repair and adaptation		
			NF-κB p65	Inflammation		
			FoxO1, FoxO3	muscle wasting		
SIRT2	Cytoplasm	Deacetylase	α-tubulin	Cellular structure	Not reported	AGK2
			PAX7	Muscle regeneration		
SIRT3	Mitochondria	Deacetylase	LCAD	FAO	NMN	3-(1H-1,2,3-triazol-4-yl) pyridine (3-TYP)
			OSCP	ATP production		
			SOD2	ROS balance		
			PDH	Energy Balance		
SIRT4	Mitochondria	Mono-ADP-ribosylase	MCD	FAO	Not reported	Not reported
		Lipoamidase	PDH	Energy Balance		
SIRT5	Mitochondria	Desuccinylase	HMGCS2	β-oxidation (FAO)	Not reported	MC3482
		Demalonylase				
		Deglutarylase				
		Deacetylase				
SIRT6	Nucleus	Deacetylase	H3K9, H3K56	Chromatin regulation	MDL801	2,4-dioxo-N-(4-(pyridin-3-yloxy)phenyl)-1,2,3,4-tetrahydroquinazoline-6-sulfonamide
			HIF-1a	Glucose uptake		
			NF-κB p65	Inflammation		
			Sox6	Muscle-fiber switch		
		Mono-ADP-ribosylase	PARP1	DNA repair		
		Deacylase	TNF-α	Modulation of cytokine signaling		
SIRT7	Nucleolus	Deacetylase	RNA Polymerase I and II (Indirect)	Histones/Ribosomal proteins	Not reported	Not reported

**Table 2:** Effects of sirtuins manipulation on skeletal muscle metabolism phenotype in rodents.

Sirtuin	Genetic manipulation	Cell targeted (rodents)	Skeletal muscle metabolism phenotype	Proposed mechanisms	Refs.
SIRT1	Conditional-knockout	Skeletal muscle (MCK-Cre, mice)	Loss of SIRT1 deacetylase activity didn't affect contraction-induced glucose uptake in EDL and soleus muscles. Glucose uptake was about 40% higher in females than males, irrespective of muscle type.	None	143
	AAV mediated-* Sirt1* overexpression	Skeletal muscle (mice)	Enhanced β-oxidation, mitochondrial biogenesis, and improved insulin signaling are insufficient in preventing obesity and systemic insulin-resistance caused by HFD.	Increased pAMPK indicates enhanced control over β-oxidation and muscle fiber composition.	17
	*In vivo* electrotransfer to overexpress SIRT1	Skeletal muscle (rats)	Attenuated HFD-induced insulin resistance, improved mitochondrial biogenesis and function, restored antioxidant enzyme activities.	Activated AMPK and PGC-1α, enhanced complex I function.	137
	Conditional-knockout	Adult skeletal muscle-specific SCs (Pax7-Cre mice).	SCs from SIRT1 muscle-specific KO mice showed no significant differences in MyoD expression or global H4K16ac levels between glucose and galactose media.	SIRT1 regulates global H4K16ac and MyoD expression in SCs under different metabolic conditions.	18
	Conditional-knockout	Skeletal muscle-specific (Myl1-Cre mice).	Decreased mitochondrial function and content, increased ROS production, higher fatigue rates during activity, with modestly improved glucose tolerance.	None	94
	Transgenic mice overexpress SIRT1	Skeletal muscle-specific (MCK-Cre mice).	Insulin-stimulated glucose uptake remains unaffected. However, 20 days of energy restriction improved insulin-stimulated glucose uptake in WT mice. No significant changes observed in body composition, energy expenditure, or substrate oxidation.	None	144
	*In vivo* electrotransfer for SIRT1 overexpression	Skeletal muscle (mice)	Upon fasting, SIRT1 overexpression prevents the induction of autophagy genes such as Bnip3, Atg4b, and Gabarapl1.	FoxO3 deacetylation and inhibition by SIRT1 lead to a decrease in autophagy in muscles during 48 hours of fasting.	61
	*In vivo* electrotransfer to overexpress mutant form H355A of SIRT1	Skeletal muscle-specific (rats)	Increased skeletal muscle mitochondrial biogenesis.	Elevated mitochondrial enzyme protein levels in rat triceps muscle tissue.	145
	Conditional-knockout	Skeletal muscle-specific (CKM-Cre mice).	Impaired muscle-specific insulin sensitivity enhancement by CR in young mice; overall glucose tolerance and insulin sensitivity unaffected under normal feeding conditions.	Sirt1 deacetylates and inactivates Stat3, potentially modulating PI3K signaling.	16
	Conditional-knockout	Skeletal muscle-specific (CKM-Cre) mice.	No impairment in mitochondrial biogenesis or PGC-1 deacetylation post voluntary wheel running or acute exercise.	None	89
	*In vivo* electrotransfer for SIRT1 overexpression	Skeletal muscle-specific (rats).	Reduced muscle mitochondrial biogenesis.	Increased SIRT1 activity downregulates mitochondrial proteins (PGC-1α, mtTFA, COX IV).	146
SIRT2	Knockout	All (mice)	Decreased muscle insulin sensitivity.	Reduction in pAkt/Akt ratio	28
SIRT3	Knockout	All (mice)	Increased insulin resistance, impaired skeletal muscle glucose uptake.	Decreased TCA cycle substrate respiration, increased fatty acid respiration.	28
	Knockout	All (mice)	Reduced muscle ATP, elevated acetylation of the ATP synthase protein and shown exercise-induced stress deficit.	SIRT3 deacetylates ATP synthase proteins, especially OSCP, enhancing mitochondrial ATP production.	55
	Knockout	All (mice)	Decreased muscle glucose oxidation, resulting in elevated lactate, pyruvate, and α-ketoglutarate levels. Insulin failed to suppress fatty acid oxidation.	Hyperacetylation of PDH E1α.	30
	Knockout	All (mice)	Impaired insulin action, decreased rate of oxygen consumption, increased oxidative stress.	Increased p-JNK, decreased IRS-1, Akt, and p-Erk.	29
	Knockout	All (mice)	51% decrease in FAO in fasting skeletal muscle.	LCAD acetylation at lysine 42.	39
	Knockout	All (mice)	Reduced mitochondrial markers and metabolic pathways.	Reduced AMPK and CREB phosphorylation, and decreased PGC-1α expression.	54
SIRT4	Knockout	All (mice)	Enhanced fatty acid oxidation, enhanced resistance to diet-induced obesity and enhanced capacity for exercise.	Increased MCD activity led to decreased amounts of malonyl CoA in skeletal muscle and white adipose tissue.	7
SIRT5	Knockout	All (mice)	Impaired β-oxidation led to medium- and long-chain acylcarnitine accumulation in liver and skeletal muscles of *Sirt5* KO mice.	Hypersuccinylation of mitochondrial proteins, including HMGCS2.	42
SIRT6	Conditional-knockout	Skeletal muscle-specific (Ckmm-Cre mice).	Dysregulated glucose homeostasis, decreased insulin sensitivity, reduced energy expenditure, and impaired exercise performance.	Reduced AMPK activity.	32
	BAC-mediated SIRT6 overexpression	All (mice)	Increased glucose uptake in gastrocnemius and soleus muscles, enhanced insulin sensitivity in gastrocnemius.	Elevated pAkt/Akt ratio in gastrocnemius muscle, increased circulating spermidine levels.	33
	Knockout	All (mice)	Exhibited lethal hypoglycemic phenotype, with increased glucose absorption in muscle and brown fat, leading to death shortly after birth.	SIRT6 inhibits HIF-1α activity by deacetylating H3K9Ac at GLUT1, LDHA, and PDHK1 promoters.	3,31
SIRT7	Knockout	All (mice)	Decreased OXPHOS complexes I (NDUFB8) and IV (MTCO1) protein levels in skeletal muscle.	None	58

**Table 3:** Skeletal muscle phenotypes in development and differentiation with modified sirtuin expression in animals.

Sirtuin	Genetic manipulation	Cell targeted (animal)	Skeletal muscle phenotype	Proposed mechanism	Refs.
SIRT1	AAV-mediated SIRT1 overexpression	Skeletal muscle-specific (mice)	Partial preservation of TA muscle mass under androgen deprivation in males.	Not reported	147
	Inducible SIRT1 overexpression	Skeletal muscle-specific (α-skeletal actin promoter in a tamoxifen-inducible manner; mice)	Increased nuclei count, smaller myonuclear domain size in muscle fibers; no impact on force capacity.	Not reported	124
	Conditional-knockout	Skeletal muscle-specific (MCK-Cre mice)	Decreased nuclei numbers and increased myonuclear domain in skeletal muscle fibers.	Not reported	124
	AAV-mediated SIRT1 overexpression	Skeletal muscle (mice)	Shift towards oxidative fiber markers increases MYHCI protein content.	Not reported	17
	Conditional-knockout	Muscle-specific knockout and satellite cells from adult skeletal muscle knockout (Pax7-Cre mice).	Reduced size initially, smaller fiber cross-sectional areas, impaired regeneration.	Increased global H4K16ac, decreased Pax7 expression.	18
	Transgenic-knockdown	Muscle-specific SIRT1 knockdown (HSA-Cre mice) subjected to two weeks of hindlimb suspension.	Increased skeletal muscle atrophy.	Not reported	92
	*In vivo* electrotransfer for SIRT1 overexpression	Skeletal muscle (mice)	Induced rapid muscle fiber hypertrophy, increasing muscle mass.	Inhibition of FoxOs and activation of PGC-1α.	61
	Conditional-knockout	Skeletal muscle-specific (Myl1-Cre mice)	No change in muscle mass.	Not reported	94
	Conditional-knockout	Skeletal muscle-specific knockout (MCK-Cre mice)	Decreased slow-twitch fiber formation, reduced OXPHOS capacity, diminished endurance.	Decreased mitochondrial electron transport gene levels.	83
	Knockout	Myoblasts collected from four-week-old wild-type & SIRT1^+/-^ (mice)	SIRT1 mutation hampers myoblast differentiation in low glucose; heterozygous mice maintain robust muscle gene expression.	Glucose restriction activates AMPK, leading to increased NAD^+^ levels and decreased NAM levels, activating SIRT1.	15
	Transgenic overexpression	All (mice)	No change in gastrocnemius muscle weight.	Not reported	148
*Sirt1AS*	AAV-mediated SIRT1AS overexpression	All (mice)	Reduced lean mass, greater muscle fiber size, and unchanged fat mass.	Enhanced Sirt1 translation via sequestering miR-34a.	105
SIRT2	Transgenic overexpression	All (mice)	Decreased muscle mass, slightly increased adiposity.	Not reported	149
	Knockout	All (mice)	Delayed muscle regeneration, exhibited muscle atrophy after injury.	Decreased MyoD, MyoG, and Myf5, increased atrogin1, and decreased IGF1 and mTOR impede muscle hypertrophy post-injury.	121
SIRT3	Knockout	All (mice)	Accelerated lean mass loss, increased protein hyper-acetylation, and expedited fiber type transition.	Triggered Ang II-induced skeletal muscle atrophy by promoting fiber-type shifting and metabolic reprogramming.	116
	Transgenic mice overexpress SIRT3M3 (Short-isoform)	Skeletal muscle-specific (FLAG with MCK, mice)	Reduced muscle mass, enhanced oxidative muscle fiber formation.	Upregulated FoxO1, leading to increased MuRF1 level.	107
	Knockout	All (mice)	No differences in muscle or fat mass observed.	Not reported	29
SIRT4	Knockout	All (mice)	No lean mass changes, unchanged muscle fiber types post-exercise, normal growth curves on a low-fat diet.	Not reported	7
SIRT5	Knockout	All (mice)	Not reported	Not reported	42
SIRT6	Conditional-knockout	Skeletal muscle-specific (SIRT6^fl/fl^ myogenin-Cre mice)	Resisted Dex-induced muscle wasting, protected against fiber type shift, and reduced atrogene expression.	SIRT6 boosts IGF/PI3K/AKT signaling via c-Jun-induced IGF2 expression.	132
	Conditional-knockout	Skeletal muscle (Myl1-Cre, mice)	Reduced oxidative fiber density, increased glycolytic fiber size.	SIRT6 influences Sox6 expression via CREB activation.	91
	Knockout	All	Decreased muscle mass, thinner fibers.	Accumulation of cytoplasmic LINE1 cDNA.	127
	CRISPR/Cas9-mediated SIRT6 knockout.	All (monkeys)	Muscle fibers predominantly comprised immature, fast-twitch fibers.	Not reported	57
	BAC-mediated SIRT6 overexpression.	All (mice)	No differences in muscle, fat mass, or body weight detected.	Not reported	33
SIRT7	Knockout	All (mice)	Not reported	Not reported	58

**Table 4:** Sirtuin modifications in diseases or muscle aging associated with skeletal muscle phenotypes in mice.

Sirtuin	Disease model/ Muscle aging	Modification	Skeletal muscle phenotypes	Proposed mechanisms	Refs.
SIRT1	Type 2 diabetes mellitus	AAV-mediated SIRT1 overexpression in Zucker diabetic fatty rats	Enhanced autophagy levels and reduced both fat deposits and swelling in skeletal muscle.	Increased LC3B, decreased p62.	150
	Cancer-induced cachexia	Reduced SIRT1 expression in cancer cells using shRNA, then implanted into mice to establish tumor growth *in vivo*	Reduced muscle fiber cross-sectional area, weaker forelimb grip strength, increased atrophy markers (Trim63 and Fbxo32 muscle-specific ubiquitin ligases), elevated ROS.	Resulted in NF-κB activation, leading to increased Nox4 expression and oxidative stress.	130
	Muscle aging (mice 26-30 months; aged)	Specific-skeletal muscles-KO (mice)	lowered exercise capacity, increased glycolytic type-IIx+IIb fibers, reduced oxidative type-IIa fibers in the soleus muscle.	Not reported	86
	Muscle aging (mice 6-12 months; middle-aged)	Specific-skeletal muscles-KO (ACTA1-Cre79Jme/J mice)	Displayed mild dystrophic features: centrally nucleated myofibers, increased fiber splitting and regeneration, muscle weakness, limited exercise endurance, and strength reduction.	Not reported	85
	Muscle aging (mice 20+ months; aged)	Skeletal muscle and satellite cell knockout using Cre-lox (B6;129-Sirt1tm1Ygu/J mice crossed with MCK-Cre and PAX7-Cre).	Decreased muscle force, fatigue resistance, and satellite cell proliferation after CTX injury.	SIRT1 enhances muscle fatigue resistance post-injury through interaction with p53.	84
	Muscle aging (mice 20+ months; aged)	Overexpressed in skeletal muscle (BAC; B6.Cg-transgenic-SIRT1-ASrn/J mice)	Increased contraction force recovery after CTX injury.	Not reported.	84
	Duchenne muscular dystrophy (mdx mice)	Skeletal muscle-specific Sirt1 overexpression (transgenic mice)	Increases slow-twitch fibers, alleviates dystrophic phenotype, and influences fiber-type plasticity.	Elevated PGC-1α, lowered muscle atrophy gene expression.	83
	Muscle aging (mice 4-10 months; middle-aged)	KO	Exhibited a phenotype resembling aging-related mitochondrial dysfunction.	Reduced mRNA levels in 13 mitochondrially encoded OXPHOS genes.	48
SIRT3	Spinal and Bulbar Muscular Atrophy (AR100Q mice)	Mice overexpress FLAG-tagged SIRT3 (M1 isoform) under the CAG promoter.	Enhanced muscle mass.	Reduced protein acetylation, particularly proteins in TCA cycle and fatty acid beta-oxidation.	131
SIRT6	Duchenne muscular dystrophy (mdx mice)	CKO; Skeletal muscle-specific (Pax7-Cre)	decreased myofiber damage, decreased dystrophic muscle pathology, and enhanced muscular performance.	SIRT6 suppresses H3K56 acetylation in the utrophin gene enhancer region, controlling its regulation.	134
	Cancer-associated cachexia (mice)	Skeletal muscle-specific SIRT6 overexpressing mice bearing tumor-induced cachexia.	Preserved muscle weight and fiber size, suppressed tumor growth.	Reduced myostatin and plasma-free fatty acids, maintained plasma insulin levels. Downregulated CXCL10 and WNT4, upregulated GLUT4 expression.	133
	Muscle aging (mice 24 months; aged)	Transgenic overexpression	Improved glucose tolerance, reduced age-related inflammation, increased exercise activity.	Upregulated AMPK	151

## Abbreviations

AAV: adeno-associated virus
Ac: acetylation
AGK2: 2-cyano-3-[5-(2,5-dichlorophenyl)-2-furanyl]-N-5-quinolinyl-2-propenamide
AICAR: 5-aminoimidazole-4-carboxamide ribonucleotide
AMPK: AMP-activated protein kinase
BAC: bacterial artificial chromosome
Bnip3: BCL2/adenovirus E1B 19 kDa proteininteracting protein-3
CREB: cAMP response element-binding protein
EDL: extensor digitorum longus
FoxOs: forkhead box Os
H4K16: histone-H4 lysine-16
HFD: high-fat diet
HIF-1α: hypoxia inducible factor-1α
KO: knockout
LCAD: long-chain acyl-CoA dehydrogenase
MCD: malonyl-CoA decarboxylase
MCK: muscle creatine kinase
MEF2C: myocyte enhancer factor-2C
Mfn1: mitofusin-1
MuRF1: Muscle RING-finger protein-1
mTORC1: mechanistic-target of rapamycin-complex 1
NAD^+^: nicotinamide adenine dinucleotide
NF-κB: nuclear factor Κb
OPA1: optic atrophy-1
OSCP: oligomycin sensitivity conferral protein
PCAF: p300/CBP-associated factor
PDH: pyruvate dehydrogenase
PGC-1α: peroxisome proliferator-activated receptor-γ coactivator-1α
PINK1: PTEN-inducible putative kinase-1
PTP1B: protein-tyrosine phosphatase-1B
SBMA: spinal and bulbar muscular atrophy
Stat3: signal transducer and activator of transcription-3

## References

[1] Baskin KK, Winders BR, Olson EN (2015). Muscle as a 'mediator' of systemic metabolism. Cell Metab.

[2] Sartori R, Romanello V, Sandri M (2021). Mechanisms of muscle atrophy and hypertrophy: implications in health and disease. Nat Commun.

[3] Mostoslavsky R, Chua KF, Lombard DB (2006). Genomic instability and aging-like phenotype in the absence of mammalian SIRT6. Cell.

[4] Imai S, Guarente L (2014). NAD+ and sirtuins in aging and disease. Trends Cell Biol.

[5] Grootaert MOJ, Bennett MR (2022). Sirtuins in atherosclerosis: guardians of healthspan and therapeutic targets. Nat Rev Cardiol.

[6] Haigis MC, Mostoslavsky R, Haigis KM (2006). SIRT4 inhibits glutamate dehydrogenase and opposes the effects of calorie restriction in pancreatic beta cells. Cell.

[7] Laurent G, German NJ, Saha AK (2013). SIRT4 coordinates the balance between lipid synthesis and catabolism by repressing malonyl CoA decarboxylase. Mol Cell.

[8] Anderson KA, Huynh FK, Fisher-Wellman K (2017). SIRT4 Is a Lysine Deacylase that Controls Leucine Metabolism and Insulin Secretion. Cell Metab.

[9] Mathias RA, Greco TM, Oberstein A (2014). Sirtuin 4 is a lipoamidase regulating pyruvate dehydrogenase complex activity. Cell.

[10] Du J, Zhou Y, Su X (2011). Sirt5 is a NAD-dependent protein lysine demalonylase and desuccinylase. Science.

[11] Mao Z, Hine C, Tian X (2011). SIRT6 promotes DNA repair under stress by activating PARP1. Science.

[12] Bheda P, Wolberger C (2013). Biochemistry: Sirtuin on a high-fat diet. Nature.

[13] Jiang H, Khan S, Wang Y (2013). SIRT6 regulates TNF-α secretion through hydrolysis of long-chain fatty acyl lysine. Nature.

[14] Guarente L (2011). Franklin H. Epstein Lecture: Sirtuins, aging, and medicine. N Engl J Med.

[15] Fulco M, Cen Y, Zhao P (2008). Glucose restriction inhibits skeletal myoblast differentiation by activating SIRT1 through AMPK-mediated regulation of Nampt. Dev Cell.

[16] Schenk S, McCurdy CE, Philp A (2011). Sirt1 enhances skeletal muscle insulin sensitivity in mice during caloric restriction. J Clin Invest.

[17] Vilà L, Roca C, Elias I (2016). AAV-mediated Sirt1 overexpression in skeletal muscle activates oxidative capacity but does not prevent insulin resistance. Mol Ther Methods Clin Dev.

[18] Ryall JG, Dell'Orso S, Derfoul A (2015). The NAD+-Dependent SIRT1 Deacetylase Translates a Metabolic Switch into Regulatory Epigenetics in Skeletal Muscle Stem Cells. Cell Stem Cell.

[19] Li Q, Zhang Q, Kim Y-R (2023). Deficiency of endothelial sirtuin1 in mice stimulates skeletal muscle insulin sensitivity by modifying the secretome. Nat Commun.

[20] Baur JA, Pearson KJ, Price NL (2006). Resveratrol improves health and survival of mice on a high-calorie diet. Nature.

[21] Cantó C, Jiang LQ, Deshmukh AS (2010). Interdependence of AMPK and SIRT1 for metabolic adaptation to fasting and exercise in skeletal muscle. Cell Metab.

[22] Feige JN, Lagouge M, Canto C (2008). Specific SIRT1 activation mimics low energy levels and protects against diet-induced metabolic disorders by enhancing fat oxidation. Cell Metab.

[23] Merrill GF, Kurth EJ, Hardie DG, Winder WW (1997). AICA riboside increases AMP-activated protein kinase, fatty acid oxidation, and glucose uptake in rat muscle. Am J Physiol.

[24] Musi N, Hirshman MF, Nygren J (2002). Metformin increases AMP-activated protein kinase activity in skeletal muscle of subjects with type 2 diabetes. Diabetes.

[25] Bonkowski MS, Sinclair DA (2016). Slowing ageing by design: the rise of NAD+ and sirtuin-activating compounds. Nat Rev Mol Cell Biol.

[26] Lan F, Cacicedo JM, Ruderman N, Ido Y (2008). SIRT1 modulation of the acetylation status, cytosolic localization, and activity of LKB1. Possible role in AMP-activated protein kinase activation. J Biol Chem.

[27] Um J-H, Park S-J, Kang H (2010). AMP-activated protein kinase-deficient mice are resistant to the metabolic effects of resveratrol. Diabetes.

[28] Lantier L, Williams AS, Hughey CC (2018). SIRT2 knockout exacerbates insulin resistance in high fat-fed mice. PLoS One.

[29] Jing E, Emanuelli B, Hirschey MD (2011). Sirtuin-3 (Sirt3) regulates skeletal muscle metabolism and insulin signaling via altered mitochondrial oxidation and reactive oxygen species production. Proc Natl Acad Sci U S A.

[30] Jing E, O'Neill BT, Rardin MJ (2013). Sirt3 regulates metabolic flexibility of skeletal muscle through reversible enzymatic deacetylation. Diabetes.

[31] Zhong L, D'Urso A, Toiber D (2010). The histone deacetylase Sirt6 regulates glucose homeostasis via Hif1alpha. Cell.

[32] Cui X, Yao L, Yang X (2017). SIRT6 regulates metabolic homeostasis in skeletal muscle through activation of AMPK. Am J Physiol Endocrinol Metab.

[33] Anderson JG, Ramadori G, Ioris RM (2015). Enhanced insulin sensitivity in skeletal muscle and liver by physiological overexpression of SIRT6. Mol Metab.

[34] Sociali G, Magnone M, Ravera S (2017). Pharmacological Sirt6 inhibition improves glucose tolerance in a type 2 diabetes mouse model. FASEB J.

[35] Gerhart-Hines Z, Rodgers JT, Bare O (2007). Metabolic control of muscle mitochondrial function and fatty acid oxidation through SIRT1/PGC-1alpha. EMBO J.

[36] Haigis MC, Sinclair DA (2010). Mammalian sirtuins: biological insights and disease relevance. Annu Rev Pathol.

[37] Garcia D, Shaw RJ (2017). AMPK: Mechanisms of Cellular Energy Sensing and Restoration of Metabolic Balance. Mol Cell.

[38] Hallows WC, Lee S, Denu JM (2006). Sirtuins deacetylate and activate mammalian acetyl-CoA synthetases. Proc Natl Acad Sci U S A.

[39] Hirschey MD, Shimazu T, Goetzman E (2010). SIRT3 regulates mitochondrial fatty-acid oxidation by reversible enzyme deacetylation. Nature.

[40] Hirschey MD, Shimazu T, Jing E (2011). SIRT3 deficiency and mitochondrial protein hyperacetylation accelerate the development of the metabolic syndrome. Mol Cell.

[41] Nasrin N, Wu X, Fortier E (2010). SIRT4 regulates fatty acid oxidation and mitochondrial gene expression in liver and muscle cells. J Biol Chem.

[42] Rardin MJ, He W, Nishida Y (2013). SIRT5 regulates the mitochondrial lysine succinylome and metabolic networks. Cell Metab.

[43] Sadhukhan S, Liu X, Ryu D (2016). Metabolomics-assisted proteomics identifies succinylation and SIRT5 as important regulators of cardiac function. Proceedings of the National Academy of Sciences.

[44] Knottnerus SJG, Bleeker JC, Wüst RCI (2018). Disorders of mitochondrial long-chain fatty acid oxidation and the carnitine shuttle. Rev Endocr Metab Disord.

[45] Civitarese AE, Carling S, Heilbronn LK (2007). Calorie restriction increases muscle mitochondrial biogenesis in healthy humans. PLoS Med.

[46] Lagouge M, Argmann C, Gerhart-Hines Z (2006). Resveratrol improves mitochondrial function and protects against metabolic disease by activating SIRT1 and PGC-1alpha. Cell.

[47] Cheng Z (2022). FoxO transcription factors in mitochondrial homeostasis. Biochem J.

[48] Gomes AP, Price NL, Ling AJY (2013). Declining NAD(+) induces a pseudohypoxic state disrupting nuclear-mitochondrial communication during aging. Cell.

[49] Zhang H-H, Ma X-J, Wu L-N (2015). SIRT1 attenuates high glucose-induced insulin resistance via reducing mitochondrial dysfunction in skeletal muscle cells. Exp Biol Med (Maywood).

[50] Howitz KT, Bitterman KJ, Cohen HY (2003). Small molecule activators of sirtuins extend Saccharomyces cerevisiae lifespan. Nature.

[51] Milne JC, Lambert PD, Schenk S (2007). Small molecule activators of SIRT1 as therapeutics for the treatment of type 2 diabetes. Nature.

[52] Koltai E, Bori Z, Osvath P (2018). Master athletes have higher miR-7, SIRT3 and SOD2 expression in skeletal muscle than age-matched sedentary controls. Redox Biol.

[53] Qiu X, Brown K, Hirschey MD, Verdin E, Chen D (2010). Calorie restriction reduces oxidative stress by SIRT3-mediated SOD2 activation. Cell Metab.

[54] Palacios OM, Carmona JJ, Michan S (2009). Diet and exercise signals regulate SIRT3 and activate AMPK and PGC-1alpha in skeletal muscle. Aging (Albany NY).

[55] Vassilopoulos A, Pennington JD, Andresson T (2014). SIRT3 deacetylates ATP synthase F1 complex proteins in response to nutrient- and exercise-induced stress. Antioxid Redox Signal.

[56] Noone J, Rochfort KD, O'Sullivan F, O'Gorman DJ (2023). SIRT4 is a regulator of human skeletal muscle fatty acid metabolism influencing inner and outer mitochondrial membrane-mediated fusion. Cell Signal.

[57] Zhang W, Wan H, Feng G (2018). SIRT6 deficiency results in developmental retardation in cynomolgus monkeys. Nature.

[58] Yoshizawa T, Sato Y, Sobuz SU (2022). SIRT7 suppresses energy expenditure and thermogenesis by regulating brown adipose tissue functions in mice. Nat Commun.

[59] Füllgrabe J, Klionsky DJ, Joseph B (2014). The return of the nucleus: transcriptional and epigenetic control of autophagy. Nat Rev Mol Cell Biol.

[60] Lee IH, Cao L, Mostoslavsky R (2008). A role for the NAD-dependent deacetylase Sirt1 in the regulation of autophagy. Proc Natl Acad Sci U S A.

[61] Lee D, Goldberg AL (2013). SIRT1 protein, by blocking the activities of transcription factors FoxO1 and FoxO3, inhibits muscle atrophy and promotes muscle growth. J Biol Chem.

[62] Mammucari C, Milan G, Romanello V (2007). FoxO3 controls autophagy in skeletal muscle in vivo. Cell Metab.

[63] Hori YS, Kuno A, Hosoda R (2011). Resveratrol ameliorates muscular pathology in the dystrophic mdx mouse, a model for Duchenne muscular dystrophy. J Pharmacol Exp Ther.

[64] Sebori R, Kuno A, Hosoda R, Hayashi T, Horio Y (2018). Resveratrol Decreases Oxidative Stress by Restoring Mitophagy and Improves the Pathophysiology of Dystrophin-Deficient mdx Mice. Oxid Med Cell Longev.

[65] Han Z, Chang C, Zhu W (2021). Role of SIRT2 in regulating the dexamethasone-activated autophagy pathway in skeletal muscle atrophy. Biochem Cell Biol.

[66] Sundaresan NR, Gupta M, Kim G, Rajamohan SB, Isbatan A, Gupta MP (2009). Sirt3 blocks the cardiac hypertrophic response by augmenting Foxo3a-dependent antioxidant defense mechanisms in mice. J Clin Invest.

[67] García-Prat L, Martínez-Vicente M, Perdiguero E (2016). Autophagy maintains stemness by preventing senescence. Nature.

[68] Choi S, Reiter DA, Shardell M (2016). 31P Magnetic Resonance Spectroscopy Assessment of Muscle Bioenergetics as a Predictor of Gait Speed in the Baltimore Longitudinal Study of Aging. J Gerontol A Biol Sci Med Sci.

[69] D'Amico D, Mottis A, Potenza F (2019). The RNA-Binding Protein PUM2 Impairs Mitochondrial Dynamics and Mitophagy During Aging. Mol Cell.

[70] Drummond MJ, Addison O, Brunker L (2014). Downregulation of E3 ubiquitin ligases and mitophagy-related genes in skeletal muscle of physically inactive, frail older women: a cross-sectional comparison. J Gerontol A Biol Sci Med Sci.

[71] Ryu D, Mouchiroud L, Andreux PA (2016). Urolithin A induces mitophagy and prolongs lifespan in C. elegans and increases muscle function in rodents. Nat Med.

[72] Askanas V, Engel WK, Nogalska A (2015). Sporadic inclusion-body myositis: A degenerative muscle disease associated with aging, impaired muscle protein homeostasis and abnormal mitophagy. Biochim Biophys Acta.

[73] Gustafsson ÅB, Dorn GW (2019). Evolving and Expanding the Roles of Mitophagy as a Homeostatic and Pathogenic Process. Physiol Rev.

[74] Liu G, Park S-H, Imbesi M (2017). Loss of NAD-Dependent Protein Deacetylase Sirtuin-2 Alters Mitochondrial Protein Acetylation and Dysregulates Mitophagy. Antioxid Redox Signal.

[75] Ma C, Zhao Y, Ding X, Gao B (2022). The role of Sirt3 in the changes of skeletal muscle mitophagy induced by hypoxic training. Gen Physiol Biophys.

[76] Wang Z, Li Q, Yang H (2023). 5-Heptadecylresorcinol Ameliorates Obesity-Associated Skeletal Muscle Mitochondrial Dysfunction through SIRT3-Mediated Mitophagy. J Agric Food Chem.

[77] Polletta L, Vernucci E, Carnevale I (2015). SIRT5 regulation of ammonia-induced autophagy and mitophagy. Autophagy.

[78] Biel TG, Lee S, Flores-Toro JA (2016). Sirtuin 1 suppresses mitochondrial dysfunction of ischemic mouse livers in a mitofusin 2-dependent manner. Cell Death Differ.

[79] Cantó C, Gerhart-Hines Z, Feige JN (2009). AMPK regulates energy expenditure by modulating NAD+ metabolism and SIRT1 activity. Nature.

[80] Suwa M, Nakano H, Radak Z, Kumagai S (2008). Endurance exercise increases the SIRT1 and peroxisome proliferator-activated receptor gamma coactivator-1alpha protein expressions in rat skeletal muscle. Metabolism.

[81] Bayod S, Del Valle J, Lalanza JF (2012). Long-term physical exercise induces changes in sirtuin 1 pathway and oxidative parameters in adult rat tissues. Exp Gerontol.

[82] Chabi B, Adhihetty PJ, O'Leary MFN, Menzies KJ, Hood DA (2009). Relationship between Sirt1 expression and mitochondrial proteins during conditions of chronic muscle use and disuse. J Appl Physiol (1985).

[83] Chalkiadaki A, Igarashi M, Nasamu AS, Knezevic J, Guarente L (2014). Muscle-specific SIRT1 gain-of-function increases slow-twitch fibers and ameliorates pathophysiology in a mouse model of duchenne muscular dystrophy. PLoS Genet.

[84] Mj M, Dl S, Aj D (2019). The role of SIRT1 in skeletal muscle function and repair of older mice. Journal of cachexia, sarcopenia and muscle.

[85] Fujiwara D, Iwahara N, Sebori R (2019). SIRT1 deficiency interferes with membrane resealing after cell membrane injury. PLoS One.

[86] Hosoda R, Kuno A, Asakura S, Horio Y (2020). Deletion of SIRT1 in the skeletal muscle decreases type IIa oxidative muscle fiber in mice. The FASEB Journal.

[87] Fritzen AM, Lundsgaard A-M, Kiens B (2020). Tuning fatty acid oxidation in skeletal muscle with dietary fat and exercise. Nat Rev Endocrinol.

[88] Gurd BJ, Perry CGR, Heigenhauser GJF, Spriet LL, Bonen A (2010). High-intensity interval training increases SIRT1 activity in human skeletal muscle. Appl Physiol Nutr Metab.

[89] Philp A, Chen A, Lan D (2011). Sirtuin 1 (SIRT1) deacetylase activity is not required for mitochondrial biogenesis or peroxisome proliferator-activated receptor-gamma coactivator-1alpha (PGC-1alpha) deacetylation following endurance exercise. J Biol Chem.

[90] Herzig S, Shaw RJ (2018). AMPK: guardian of metabolism and mitochondrial homeostasis. Nat Rev Mol Cell Biol.

[91] Song M-Y, Han CY, Moon YJ, Lee JH, Bae EJ, Park B-H (2022). Sirt6 reprograms myofibers to oxidative type through CREB-dependent Sox6 suppression. Nat Commun.

[92] Mercken EM, Mitchell SJ, Martin-Montalvo A (2014). SRT2104 extends survival of male mice on a standard diet and preserves bone and muscle mass. Aging Cell.

[93] Kong X, Wang R, Xue Y (2010). Sirtuin 3, a new target of PGC-1alpha, plays an important role in the suppression of ROS and mitochondrial biogenesis. PLoS One.

[94] Menzies KJ, Singh K, Saleem A, Hood DA (2013). Sirtuin 1-mediated Effects of Exercise and Resveratrol on Mitochondrial Biogenesis. J Biol Chem.

[95] Shi L, Zhang Y, Zhang J (2021). MiR-339 is a potential biomarker of coronary heart disease to aggravate oxidative stress through Nrf2/FOXO3 targeting Sirt2. Ann Palliat Med.

[96] Nakazawa H, Chang K, Shinozaki S (2017). iNOS as a Driver of Inflammation and Apoptosis in Mouse Skeletal Muscle after Burn Injury: Possible Involvement of Sirt1 S-Nitrosylation-Mediated Acetylation of p65 NF-κB and p53. PLoS One.

[97] Mitchell SJ, Martin-Montalvo A, Mercken EM (2014). The SIRT1 activator SRT1720 extends lifespan and improves health of mice fed a standard diet. Cell Rep.

[98] Mounier R, Théret M, Arnold L (2013). AMPKα1 regulates macrophage skewing at the time of resolution of inflammation during skeletal muscle regeneration. Cell Metab.

[99] Sousa-Victor P, García-Prat L, Muñoz-Cánoves P (2022). Control of satellite cell function in muscle regeneration and its disruption in ageing. Nat Rev Mol Cell Biol.

[100] Rathbone CR, Booth FW, Lees SJ (2009). Sirt1 increases skeletal muscle precursor cell proliferation. Eur J Cell Biol.

[101] Wang L, Xue R, Sun C (2016). SIRT 1 regulates C2C12 myoblast cell proliferation by activating Wnt signaling pathway. Int J Clin Exp Med.

[102] Wang L, Zhang T, Xi Y, Yang C, Sun C, Li D (2016). Sirtuin 1 promotes the proliferation of C2C12 myoblast cells via the myostatin signaling pathway. Mol Med Rep.

[103] Wu G, Song C, Lu H (2014). Sirt2 induces C2C12 myoblasts proliferation by activation of the ERK1/2 pathway. Acta Biochim Biophys Sin (Shanghai).

[104] Sincennes M-C, Brun CE, Lin AYT (2021). Acetylation of PAX7 controls muscle stem cell self-renewal and differentiation potential in mice. Nat Commun.

[105] Wang G, Wang Y, Xiong Y (2016). Sirt1 AS lncRNA interacts with its mRNA to inhibit muscle formation by attenuating function of miR-34a. Sci Rep.

[106] Fulco M, Schiltz RL, Iezzi S (2003). Sir2 regulates skeletal muscle differentiation as a potential sensor of the redox state. Mol Cell.

[107] Lin L, Chen K, Abdel Khalek W (2014). Regulation of skeletal muscle oxidative capacity and muscle mass by SIRT3. PLoS One.

[108] Dumont NA, Bentzinger CF, Sincennes M-C, Rudnicki MA (2015). Satellite Cells and Skeletal Muscle Regeneration. Compr Physiol.

[109] Zhao X, Sternsdorf T, Bolger TA, Evans RM, Yao T-P (2005). Regulation of MEF2 by histone deacetylase 4- and SIRT1 deacetylase-mediated lysine modifications. Mol Cell Biol.

[110] Amat R, Planavila A, Chen SL, Iglesias R, Giralt M, Villarroya F (2009). SIRT1 controls the transcription of the peroxisome proliferator-activated receptor-gamma Co-activator-1alpha (PGC-1alpha) gene in skeletal muscle through the PGC-1alpha autoregulatory loop and interaction with MyoD. J Biol Chem.

[111] Abdel Khalek W, Cortade F, Ollendorff V (2014). SIRT3, a mitochondrial NAD^+^-dependent deacetylase, is involved in the regulation of myoblast differentiation. PLoS One.

[112] Nedachi T, Kadotani A, Ariga M, Katagiri H, Kanzaki M (2008). Ambient glucose levels qualify the potency of insulin myogenic actions by regulating SIRT1 and FoxO3a in C2C12 myocytes. Am J Physiol Endocrinol Metab.

[113] Ljubicic V, Burt M, Lunde JA, Jasmin BJ (2014). Resveratrol induces expression of the slow, oxidative phenotype in mdx mouse muscle together with enhanced activity of the SIRT1-PGC-1α axis. Am J Physiol Cell Physiol.

[114] Wen W, Chen X, Huang Z (2020). Resveratrol regulates muscle fiber type conversion via miR-22-3p and AMPK/SIRT1/PGC-1α pathway. J Nutr Biochem.

[115] J B, Z L, Jj J (2010). Characterization of the murine SIRT3 mitochondrial localization sequence and comparison of mitochondrial enrichment and deacetylase activity of long and short SIRT3 isoforms. Journal of cellular biochemistry.

[116] Zheng J, Gao J, Zhang Q, Wu X, Shen W, Sun M (2020). Sirtuin 3 deficiency accelerates Angiotensin II-induced skeletal muscle atrophy. Connect Tissue Res.

[117] Yucel N, Wang YX, Mai T (2019). Glucose Metabolism Drives Histone Acetylation Landscape Transitions that Dictate Muscle Stem Cell Function. Cell Rep.

[118] Cerletti M, Jang YC, Finley LWS, Haigis MC, Wagers AJ (2012). Short-term calorie restriction enhances skeletal muscle stem cell function. Cell Stem Cell.

[119] Mañas-García L, Guitart M, Duran X, Barreiro E (2020). Satellite Cells and Markers of Muscle Regeneration during Unloading and Reloading: Effects of Treatment with Resveratrol and Curcumin. Nutrients.

[120] Niu W, Wang H, Wang B, Mao X, Du M (2021). Resveratrol improves muscle regeneration in obese mice through enhancing mitochondrial biogenesis. J Nutr Biochem.

[121] Lee E-J, Lee M-M, Park S, Jeong K-S (2022). Sirt2 positively regulates muscle regeneration after Notexin-induced muscle injury. Exp Mol Pathol.

[122] Gombos Z, Koltai E, Torma F (2021). Hypertrophy of Rat Skeletal Muscle Is Associated with Increased SIRT1/Akt/mTOR/S6 and Suppressed Sestrin2/SIRT3/FOXO1 Levels. Int J Mol Sci.

[123] Koltai E, Bori Z, Chabert C (2017). SIRT1 may play a crucial role in overload-induced hypertrophy of skeletal muscle. J Physiol.

[124] Ross JA, Levy Y, Svensson K, Philp A, Schenk S, Ochala J (2018). SIRT1 regulates nuclear number and domain size in skeletal muscle fibers. J Cell Physiol.

[125] Eisele PS, Salatino S, Sobek J, Hottiger MO, Handschin C (2013). The peroxisome proliferator-activated receptor γ coactivator 1α/β (PGC-1) coactivators repress the transcriptional activity of NF-κB in skeletal muscle cells. J Biol Chem.

[126] Sandri M, Lin J, Handschin C (2006). PGC-1alpha protects skeletal muscle from atrophy by suppressing FoxO3 action and atrophy-specific gene transcription. Proc Natl Acad Sci U S A.

[127] Simon M, Van Meter M, Ablaeva J (2019). LINE1 Derepression in Aged Wild-Type and SIRT6-Deficient Mice Drives Inflammation. Cell Metab.

[128] Zhang Y, Wang J, Wang X (2020). The autophagic-lysosomal and ubiquitin proteasome systems are simultaneously activated in the skeletal muscle of gastric cancer patients with cachexia. Am J Clin Nutr.

[129] Huang Y, Zhu X, Chen K (2019). Resveratrol prevents sarcopenic obesity by reversing mitochondrial dysfunction and oxidative stress via the PKA/LKB1/AMPK pathway. Aging (Albany NY).

[130] Dasgupta A, Shukla SK, Vernucci E (2020). SIRT1-NOX4 signaling axis regulates cancer cachexia. J Exp Med.

[131] Garcia Castro DR, Mazuk JR, Heine EM (2023). Increased SIRT3 combined with PARP inhibition rescues motor function of SBMA mice. iScience.

[132] Mishra S, Cosentino C, Tamta AK (2022). Sirtuin 6 inhibition protects against glucocorticoid-induced skeletal muscle atrophy by regulating IGF/PI3K/AKT signaling. Nat Commun.

[133] Samant SA, Pillai VB, Gupta MP (2021). Skeletal muscle-specific over-expression of the nuclear sirtuin SIRT6 blocks cancer-associated cachexia by regulating multiple targets. JCSM Rapid Commun.

[134] Georgieva AM, Guo X, Bartkuhn M (2022). Inactivation of Sirt6 ameliorates muscular dystrophy in mdx mice by releasing suppression of utrophin expression. Nat Commun.

[135] Fröjdö S, Durand C, Molin L (2011). Phosphoinositide 3-kinase as a novel functional target for the regulation of the insulin signaling pathway by SIRT1. Mol Cell Endocrinol.

[136] Sun C, Zhang F, Ge X (2007). SIRT1 improves insulin sensitivity under insulin-resistant conditions by repressing PTP1B. Cell Metab.

[137] Zhang H-H, Qin G-J, Li X-L (2015). SIRT1 overexpression in skeletal muscle in vivo induces increased insulin sensitivity and enhanced complex I but not complex II-V functions in individual subsarcolemmal and intermyofibrillar mitochondria. J Physiol Biochem.

[138] Szendroedi J, Phielix E, Roden M (2012). The role of mitochondria in insulin resistance and type 2 diabetes mellitus. Nat Rev Endocrinol.

[139] Liang F, Chen R, Nakagawa A (2011). Low-Frequency Electroacupuncture Improves Insulin Sensitivity in Obese Diabetic Mice through Activation of SIRT1/PGC-1α in Skeletal Muscle. Evid Based Complement Alternat Med.

[140] Arora A, Dey CS (2014). SIRT2 negatively regulates insulin resistance in C2C12 skeletal muscle cells. Biochim Biophys Acta.

[141] Song Y, Shi J, Wu Y (2014). Metformin ameliorates insulin resistance in L6 rat skeletal muscle cells through upregulation of SIRT3. Chin Med J (Engl).

[142] Yechoor VK, Patti M-E, Ueki K (2004). Distinct pathways of insulin-regulated versus diabetes-regulated gene expression: an in vivo analysis in MIRKO mice. Proc Natl Acad Sci U S A.

[143] Jh K, Je P, J D (2021). Sirtuin 1 is not required for contraction-stimulated glucose uptake in mouse skeletal muscle. Journal of applied physiology (Bethesda, Md: 1985).

[144] White AT, McCurdy CE, Philp A, Hamilton DL, Johnson CD, Schenk S (2013). Skeletal muscle-specific overexpression of SIRT1 does not enhance whole-body energy expenditure or insulin sensitivity in young mice. Diabetologia.

[145] Higashida K, Kim SH, Jung SR, Asaka M, Holloszy JO, Han D-H (2013). Effects of resveratrol and SIRT1 on PGC-1α activity and mitochondrial biogenesis: a reevaluation. PLoS Biol.

[146] Gurd BJ, Yoshida Y, Lally J, Holloway GP, Bonen A (2009). The deacetylase enzyme SIRT1 is not associated with oxidative capacity in rat heart and skeletal muscle and its overexpression reduces mitochondrial biogenesis. J Physiol.

[147] Gordon BS, Burns PK, Laskin GR (2023). SIRT1 induction in the skeletal muscle of male mice partially preserves limb muscle mass but not contractile force in response to androgen deprivation. J Physiol.

[148] Bordone L, Cohen D, Robinson A (2007). SIRT1 transgenic mice show phenotypes resembling calorie restriction. Aging Cell.

[149] Le W, Ce F, Nl B (2023). SIRT2 transgenic over-expression does not impact lifespan in mice. Aging cell.

[150] Mao Z-J, Xia W-S, Chai F (2021). Yunpi Heluo decoction attenuates insulin resistance by regulating SIRT1-FoxO1 autophagy pathway in skeletal muscle of Zucker diabetic fatty rats. J Ethnopharmacol.

[151] Roichman A, Kanfi Y, Glazz R (2017). SIRT6 Overexpression Improves Various Aspects of Mouse Healthspan. J Gerontol A Biol Sci Med Sci.

